# Performance Analysis of Active Structural Acoustic Control Applied to a Washing Machine

**DOI:** 10.3390/s22197357

**Published:** 2022-09-28

**Authors:** Stanislaw Wrona

**Affiliations:** Department of Measurements and Control Systems, Silesian University of Technology, Akademicka 16, 44-100 Gliwice, Poland; stanislaw.wrona@polsl.pl

**Keywords:** active noise control, active casing, structural control, device noise control

## Abstract

Great efforts are undertaken by scientists and manufacturers to reduce noise generated by devices present in the human environment. This task is particularly difficult in the case of low-frequency noise; however, active noise reduction methods can be a solution to such problems. The purpose of this article is to present the active noise-controlling casing method applied to a washing machine, whose noise generated during the spinning process is to be reduced. The paper presents a set of experimental measurements and analyses. It also provides hardware and software configuration details. The employed active control system can efficiently reduce noise, even by 16 dBA in the low-frequency range up to 300 Hz, which is an important step towards making the technology ready for manufacturing. The conclusions drawn are valid for different active noise-control applications, considering a variety of devices.

## 1. Introduction

Noise exposure constitutes a serious threat to human health and comfort [1,2]. High sound-pressure levels may cause hearing loss, induce stress, cause voice communication problems, etc.; however, even moderate noise endangers our nervous system and makes it difficult to work or rest. Thus, noise reduction has gained attention from both researchers and manufacturers. The most feasible approach is to reduce the noise level at the source. However, passive sound-absorbing materials commonly used for this purpose have many limitations. They have poor performance for low frequencies. The barrier must be too thick (and in many cases also too heavy) for practical usage. Such limitations have influenced interest in active systems, which may be very effective for low frequencies and complement passive methods in their weak points [3,4,5]. A number of single-panel solutions have been developed in recent years [6,7,8], where vibrations of a thin barrier were controlled by actuators in order to reduce noise transmission and radiation. However, the control of 3-dimensional shells and enclosures is much more demanding. The idea of using whole casings as active barriers was initially proposed by Fuller et al. [9]. A similar idea was later developed by other researchers [10,11,12].

The aim of this work is to apply and evaluate the active casing method adapted to a washing machine. In the first step, preliminary acoustic measurements of the washing machine were made. This investigation served the need to better understand the nature of the noise generated by the appliance and to determine a beneficial actuators arrangement. The measurements performed are presented in Section 2. In the second step, the active control experiments have been performed. The employed control system and its performance is described in Section 3. The noise-reduction results are analyzed and discussed based on frequency analysis. The achieved substantial noise reduction levels paves the way for the future commercial applications. The conclusions drawn are summarized in Section 4.

## 2. Preliminary Measurements

### 2.1. Laboratory Setup

**Figure 1:**
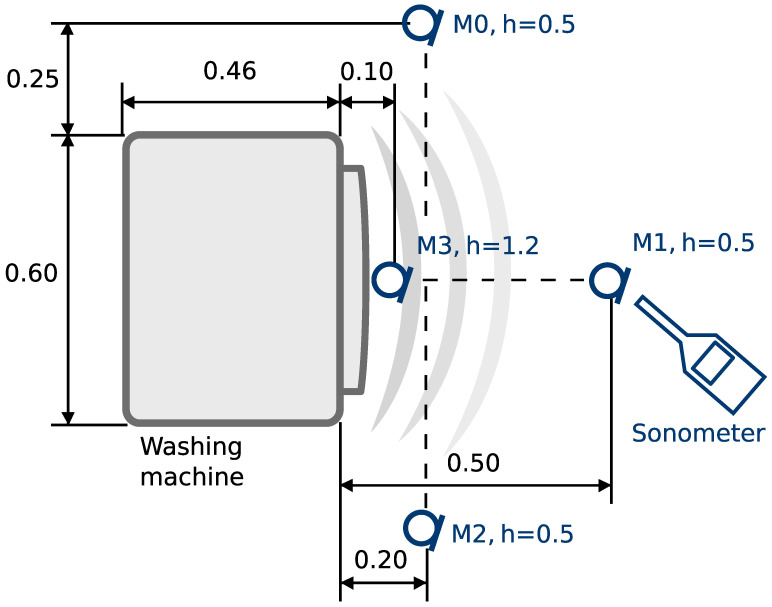
A scheme of the measurement system. All dimensions are given in (m).

The laboratory setup consisted of four monitoring microphones Beyerdynamic MM1 [13] (cf. Figure 1) and a certified sonometer SVAN 912 AE [14]. The microphones and the sonometer were kept at the same locations for all measurements series. The acoustic measurements were performed for three drum loadings (no unbalanced load, loading of 300 g and loading of 400 g) and a range of spinning velocities (up to 1400 RPM, incremented by 100 RPM). The unbalanced load was realized with metal weights attached with magnets to the drum. The reason for applying such an unbalanced load was to mimic the real loading of the washing machine while maintaining the repeatability of the experiments.

### 2.2. Results

**Figure 2:**
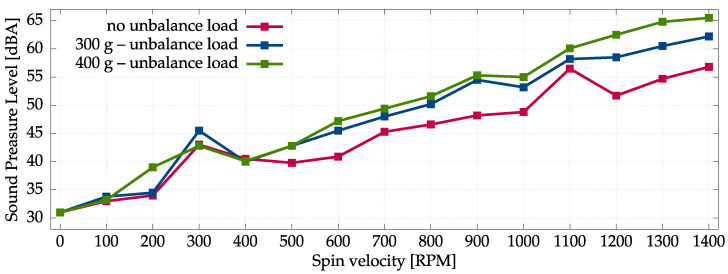
Sound Pressure Level measured at the M1 location using a certified sonometer.

**Figure 3:**
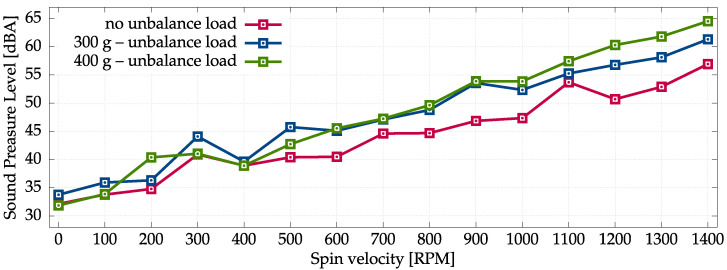
Average Sound-Pressure Level measured at M0–M3 locations using four measurement microphones.

The aggregated results of all acoustic measurement series are presented in Figure 2 and Figure 3. The measurements performed with sonometer and measurement microphones are very consistent. It follows from analysis that increased unbalanced loading results in increased sound pressure level of noise generated by the washing machine, especially for higher spinning velocities. On the other hand, the sound-pressure level of noise is also strictly correlated with the spinning velocities—increased velocity results in increased noise. In fact, the noise generated by the washing machine outside the spinning phase is rather mild. These results are consistent with intuitive expectations.

**Figure 4:**
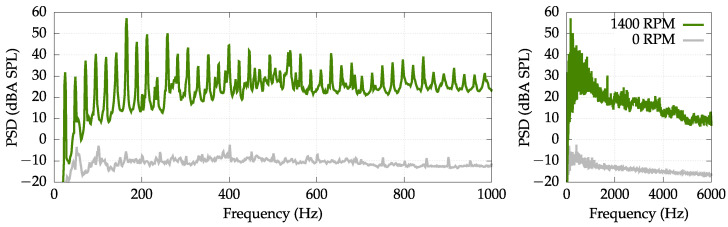
PSD with an A-weighting measured at M0–M3 locations using four measurement microphones. Unbalanced load of 400 g. Spin velocity equal to 1400 RPM.

The performed measurements, however, also served the need to provide an insight into the frequency spectrum of the generated noise. The exemplary frequency analysis of measurements obtained for the 400 g unbalance load and the highest considered spinning velocity is presented in Figure 4. An important conclusion can be drawn from Figure 4 that a majority of the acoustic energy emitted by the appliance is contained in the low frequency band (below 1000 Hz, and the highest peaks are limited to even lower-frequency bands up to 600 Hz). It is an important factor when considering an application of active control methods because it means that the active control (which performs best for low frequencies) can be especially beneficial for the considered device.

It is also noteworthy that the generated noise has a strong multitonal nature—the generated noise consists of fundamental tone related to the spinning velocity (e.g., 23.3 Hz for 1400 RPM spinning velocity) and numerous harmonic frequencies. This kind of noise is preferred when applying the active control solution, because it facilitates the performance of the adaptive control algorithm (algorithm can predict future noise samples even if the reference signal does not provide information about the noise with sufficient advance in time). Both of the aforementioned observations support the conclusion that active control might be a well-suited solution for the considered noise problem.

## 3. Active Control Experiments

### 3.1. Laboratory Setup

The adopted control approach is based on Active Structural Acoustic Control (ASAC), which alters the vibration of a barrier in order to reduce noise transmitted through it. Although this approach stems from individual single-panel barriers, in recent years it has been studied in applications for whole three-dimensional casings [12].

**Figure 5:**
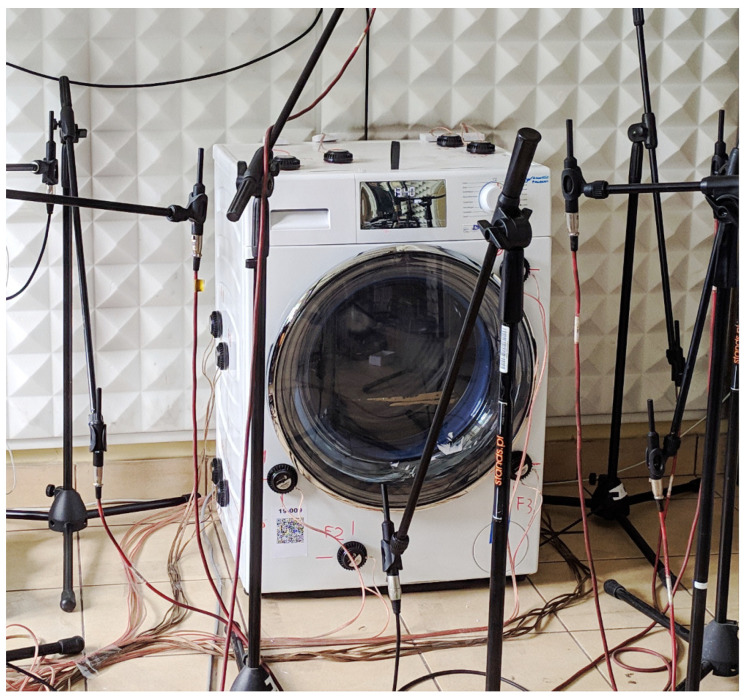
A photograph of the active control laboratory setup.

The utilized control system employed 16 inertial actuators (Dayton Audio DAEX32EP-4 [15]) attached to the washing machine casing according to an arrangement optimization method [16,17] (4 actuators per controlled wall). The aim of the optimization was to maximize the controllability measures of each wall. Detailed locations of the actuators are given in Table 1. Moreover, 9 error microphones and 1 reference microphone were used. The error microphones have been distributed around the casing in a distance of approximately 0.5 m from the appliance (cf. Figure 5; in a final application they will be substituted by structural sensors, e.g., accelerometers, using the Virtual Microphone Control approach [18,19]). The reference microphone for the feed-forward control system was placed in a gap between the back of the washing machine and a wall, at which the appliance was located (in a final application, it will be located inside the casing or the reference signal will be synthesised based on drum-velocity measurements). As a control system, 4 dSPACE DS1104 boards were used. They provided a sufficient computational power together with a large number of available analogue inputs and outputs, as the dimensions of the control system were 16 × 9.

### 3.2. Active Control Algorithm

The control algorithm was based on the time-domain FxLMS (Filtered-x Least Mean Square) control scheme [12]. The algorithm is commonly utilized to provide adaptation in many active control applications. Its advantages are, e.g., the simplicity of implementation, robustness, and the low complexity of computations [20]. What is specific to this control system is that the vibration actuators are used as secondary sources to directly reduce the noise monitored by the error microphones. The way that the actuators affect the casing vibrations depends on the distribution of error microphones. If there were only a few error microphones sparsely distributed (the distance between microphones would be much greater than half of the noise wave length), then the casing would act as a distributed speaker (structural sound source) generating local zones of quiet around the error microphones. However, if the error microphones are more densely surrounding the structure, with a distance between microphones not greater than half of the noise wave length, then the phenomenon would be different and global noise reduction would be achieved. The actuators then alter the vibration distribution in order to reduce the noise radiation. What has been observed is that the vibration amplitude with active control can be even higher than without it, but the noise radiation is globally reduced. It is an important conclusion from the energy effort point of view—to achieve a global noise reduction, the vibrations of the casing do not have to be entirely suppressed, they only have to be appropriately altered.

**Figure 6:**
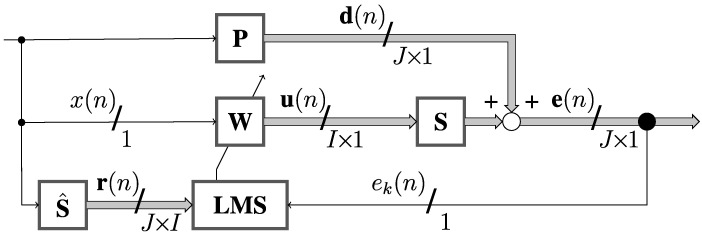
Multi-channel control system with the Switched-error FxLMS algorithm.

Due to strong couplings between all actuators and error microphones, a complete multiple-input multiple-output (MIMO) 16×9 system must be used. However, a usual MIMO FxLMS implementation would require too much computational power. Instead, a Switched-Error FxLMS algorithm modification has been used [21]. It reduces the computational complexity as at the time the adaptation is performed according to only a single error signal (cf. Figure 6). This approach slows down the convergence but provides the same steady-state noise reduction levels as a usual full MIMO system.

In Figure 6, the symbol W is the adaptive control filters vector (of dimension I×1, where *I* is the number of actuators, equal to 16 in this research), and P is the primary paths vector (of dimension J×1, where *J* is the number of error sensors, equal to 9 in this paper), defined between the reference and error sensors. S stands for the secondary paths matrix of dimension J×I defined between the inputs of the actuators and outputs of the error sensors. The symbol $\hat{S}$ stands for the secondary path model. In turn, x(n) is the estimated scalar reference signal, r(n) is the filtered-reference signals matrix of dimension J×I, and u(n) is the control signals vector of dimension I×1. Further, signals d(n) and e(n) are the primary disturbances vector and the error signals vector, respectively, both of dimension J×1, at positions of the error sensors where noise reduction is desired. Signal $e_{k}\left(n\right)$ is the *k*th selected error signal currently employed for adaption and cyclically changed as described in [21].

The sampling frequency was equal to 2 kHz. The cut-off frequency of low pass filters was 800 Hz. The number of parameters in the secondary path models S (FIR filters) was equal to 128 and was selected based on impulse responses of the secondary paths. The adopted control filter W length was 256. The Normalized Leaky LMS algorithm step size was 0.001, and the leak factor was 0.0001, and they were selected based on the performance of the control system.

### 3.3. Results

**Figure 7:**
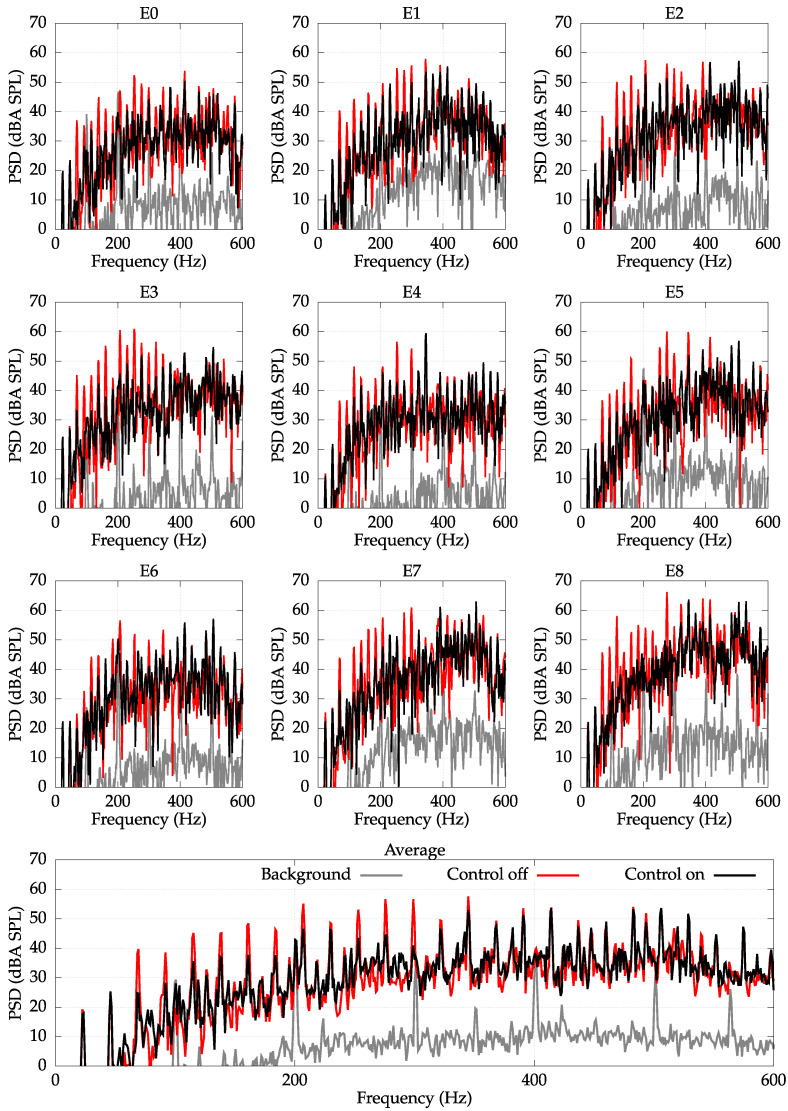
PSD with an A-weighting measured at error microphones E0–E8. Unbalanced load of 500 g. Spin velocity 1340 RPM. Average reduction: 4 dBA.

The control experiments were performed for the unbalanced load of 500 g and spinning velocity equal to 1340 RPM. The frequency spectra of signals obtained with error microphones with and without control are presented in Figure 7.

It follows from an analysis of Figure 7 that all frequency components in the range from 50 Hz to 300 Hz are significantly reduced. The initial two frequencies of 23 Hz and 47 Hz were not reduced due to the fact that the employed actuators do not transmit the control signals in this frequency range; however, these frequencies are barely noticeable by a human ear (they are very weak considering the A-weighting). On the other hand, frequencies higher than 300 Hz are beyond the assumed frequency range of operation for the employed controlled system—only some slight reduction can be noticed.

**Figure 8:**
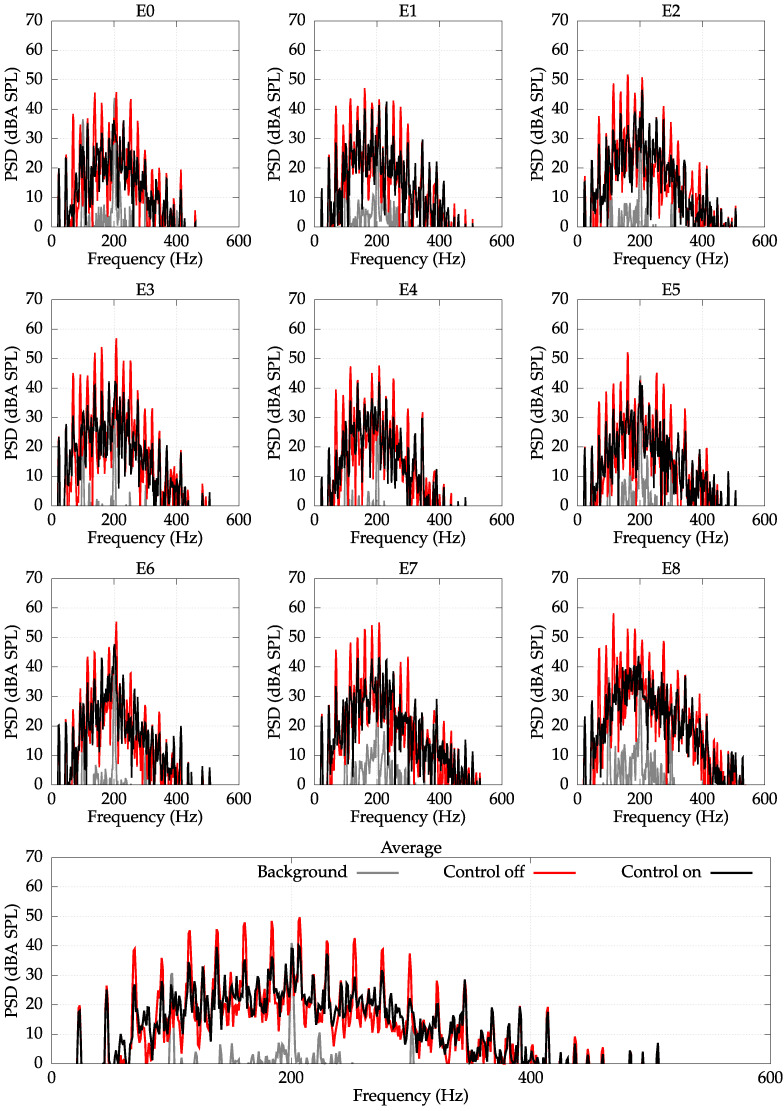
PSD with an A-weighting measured at error microphones E0–E8. Unbalanced load of 500 g. Spin velocity of 1340 RPM. The signals are filtered with a lowpass filter of cutoff frequency $f_{c}=300\;\mathrm{Hz}$. Average reduction: 16 dBA.

Due to the fact that the higher-frequency components were mostly unreduced, the average reduction for whole frequency range reached only 4 dBA. However, if the error signals would be filtered by a lowpass filter of cut-off frequency equal to 300 Hz (cf. Figure 8), the average reduction reached 16 dBA, which is a significant level of noise reduction.

**Figure 9:**
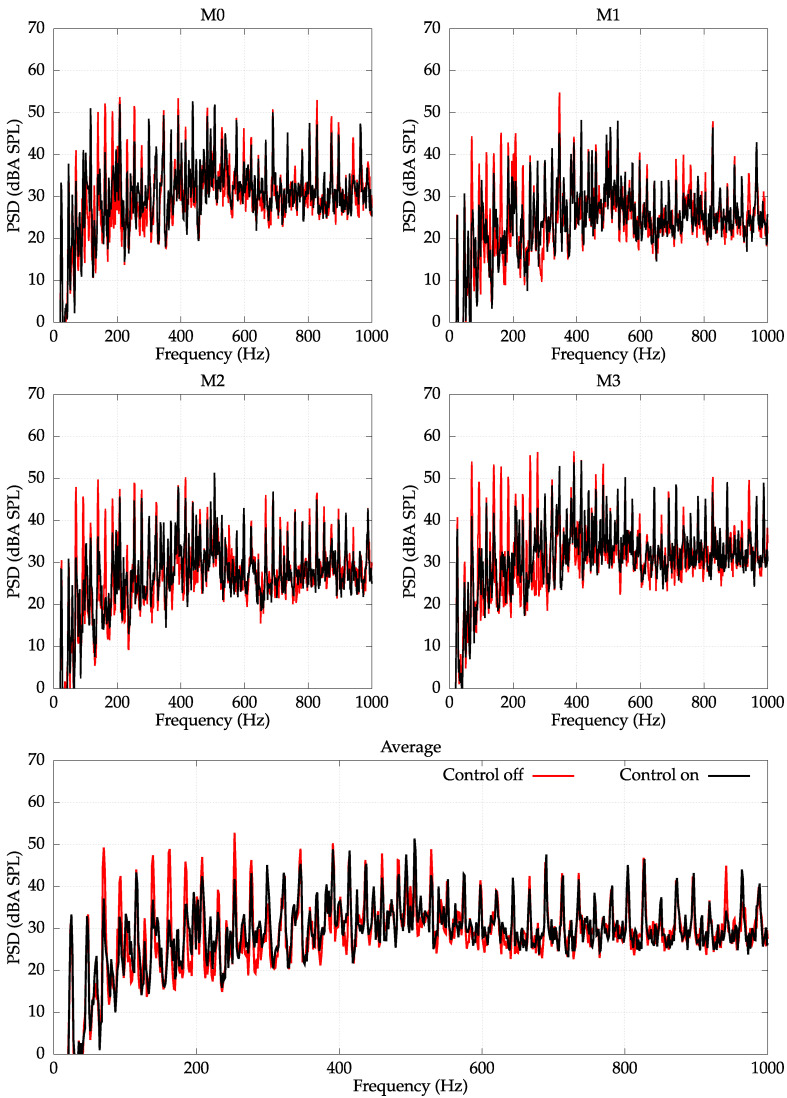
PSD with an A-weighting measured at monitoring microphones M0–M3. Unbalance load of 500 g. Spin velocity 1340 RPM. Average reduction: 3 dBA.

In addition to error microphones, four monitoring microphones were used to observe noise-reduction levels at a greater distance from the washing machine. These microphones were not used by the control system, and their signals were acquired using 96 kHz sampling frequency. The frequency spectra of signals obtained with monitoring microphones with and without control are presented in Figure 9. The noise-reduction results observed by monitoring microphones resembles the results observed by error microphones: the control system performs well in the frequency range up to approximately 300 Hz; however, there are still considerable frequency components that are unreduced in the bandwidth approximately up to 600 Hz.

**Figure 10:**
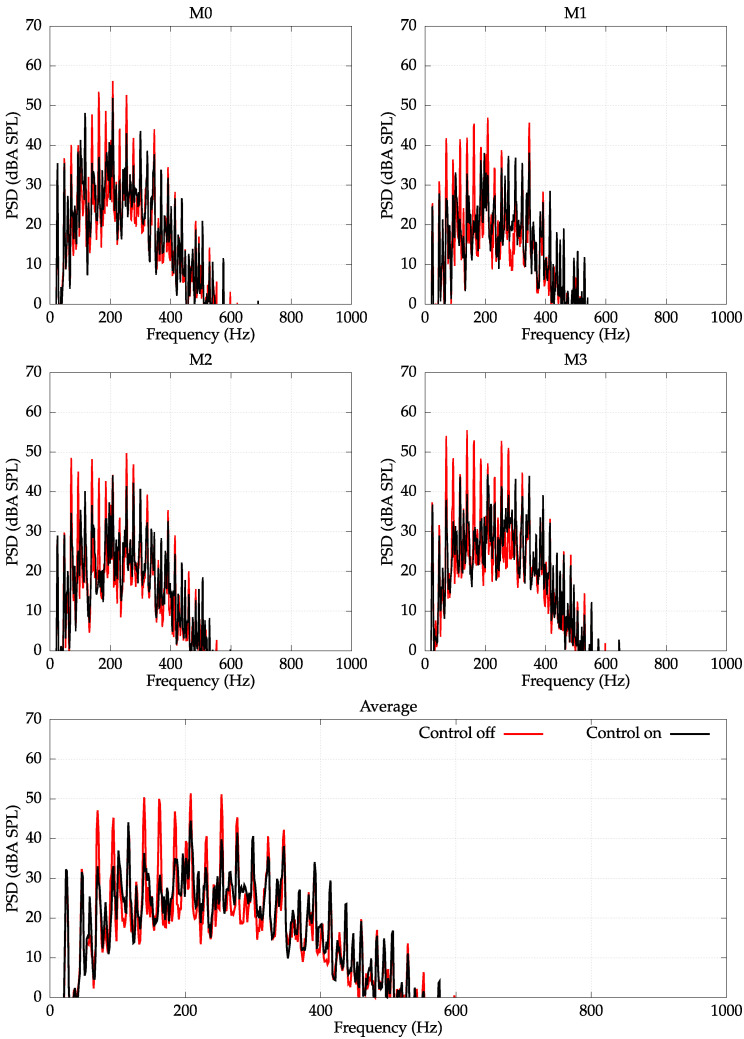
PSD with an A-weighting measured at monitoring microphones M0–M3. Unbalanced load of 500 g. Spin velocity 1340 RPM. The signals are filtered with a lowpass filter of cutoff frequency $f_{c}=300\;\mathrm{Hz}$. Average reduction: 6 dBA.

The average reduction observed by monitoring microphones reached 3 dBA in the whole frequency range and 6 dBA in the frequency range limited to 300 Hz (cf. Figure 10). However, if the control system after a further development process could extend its frequency range of operation to approximately 500–600 Hz, the average reduction in the whole audible frequency range would be significantly increased. Moreover, an active control system with an extended frequency range could reach a point at which it could be complemented with a felt or other passive sound-absorbing material, making a comprehensive noise control solution. Alternatively, the gap between active system and the passive sound absorbing materials could be filled by acoustic metamaterials designed specifically for this purpose.

## 4. Conclusions

In this research, preliminary acoustic measurements of the washing machine were performed and subsequently analysed. Then, active control experiments were performed. The obtained noise-reduction results are presented and discussed.

The main conclusion of the presented work is that the employed active control system can efficiently reduce noise by 16 dB in the low-frequency range up to 300 Hz. Although the components of the control system were described in the literature in the past, such high levels of noise reduction in near-real conditions are obtained for the first time. This is a significant achievement, taking into account a global character of the noise reduction (in the whole room) and the real operating conditions of the washing machine. It is also an important step towards making the technology ready for manufacturing.

However, to achieve reduction in the whole audible frequency range, the frequency range of the control system operation should be extended to approximately 500–600 Hz. Therefore, the control system should be further developed, including, among other things, an increase in the sampling frequency. An active control system with an extended frequency range of operation could reach a point at which it could be complemented with a felt or other passive sound absorbing material, making a comprehensive noise control solution that could be easily commercialized. Acoustic metamaterials should also be considered as a potential solution to fill the gap between active and passive means.

It is also noteworthy that the control system and its sensors, apart from noise reduction, can enable a wide range of auto-diagnostics for the appliance (e.g., the detection of damaged bearings or other components). In the case of detection of any malfunction, the user can be warned before a severe failure of the appliance occurs.

## Figures and Tables

**Table 1:** Detailed locations of the actuators attached to the casing. The origin of the coordinates system is in the left bottom corner of each wall.

	Left Wall		Right Wall		Top Wall		Front Wall	
No.	x (m)	y (m)	x (m)	y (m)	x (m)	y (m)	x (m)	y (m)
1	0.24	0.48	0.22	0.48	0.11	0.08	0.51	0.68
2	0.36	0.52	0.10	0.52	0.21	0.19	0.08	0.18
3	0.26	0.13	0.20	0.13	0.48	0.36	0.18	0.09
4	0.39	0.16	0.07	0.16	0.50	0.26	0.53	0.17

## Data Availability

The data presented in this study are available on request from the corresponding author.
